# Modeling Indoor Relative Humidity and Wood Moisture Content as a Proxy for Wooden Home Fire Risk

**DOI:** 10.3390/s19225050

**Published:** 2019-11-19

**Authors:** Torgrim Log

**Affiliations:** Department of Safety, Chemistry and Biomedical Laboratory Sciences, Fire Disaster Research Group, Western Norway University of Applied Sciences, 5528 Haugesund, Norway; torgrim.log@hvl.no; Tel.: +47-90-0500-01

**Keywords:** relative humidity, dry wood, fire risk, conflagration risk, modeling

## Abstract

Severe wooden home conflagrations have previously been linked to the combination of very dry indoor climate in inhabited buildings during winter time, resulting in rapid fire development and strong winds spreading the fire to neighboring structures. Knowledge about how ambient conditions increase the fire risk associated with dry indoor conditions is, however, lacking. In the present work, the moisture content of indoor wooden home wall panels was modeled based on ambient temperature and relative humidity recorded at meteorological stations as the climatic boundary conditions. The model comprises an air change rate based on ambient and indoor (22 °C) temperatures, indoor moisture sources and wood panel moisture sorption processes; it was tested on four selected homes in Norway during the winter of 2015/2016. The results were compared to values recorded by indoor relative humidity sensors in the homes, which ranged from naturally ventilated early 1900s homes to a modern home with balanced ventilation. The modeled indoor relative humidity levels during cold weather agreed well with recorded values to within 3% relative humidity (RH) root mean square deviation, and thus provided reliable information about expected wood panel moisture content. This information was used to assess historic single home fire risk represented by an estimated time to flashover during the studied period. Based on the modelling, it can be concluded that three days in Haugesund, Norway, in January 2016 were associated with very high conflagration risk due to dry indoor wooden materials and strong winds. In the future, the presented methodology may possibly be based on weather forecasts to predict increased conflagration risk a few days ahead. This could then enable proactive emergency responses for improved fire disaster risk management.

## 1. Introduction

Fires represent a threat worldwide. Fires in cars, for example, develop when non-hygroscopic plastic-based products or fuel is involved in combustion. These fires generally develop very fast [1]. Modern products are in general more dangerous than products used in previous periods [2]. This is also the case for industry processing hydrocarbons. Initiatives are therefore taken to detect the fire risk early, that is, before leaks can develop into dangerous situations [3]. Homes may also be involved in fires, and in several areas homes are traditionally made of wood based materials. While concrete dominated the home constructions for many years, the emerging focus on sustainability increases the popularity of wood constructions including sustainable safety measures [4].

In Norway, there is a rich tradition regarding wooden constructions, which dominate many town centers and suburban detached home developments. This stems from the availability of high-quality lumber from abundant forests covering large lowland areas, including areas also north of the Arctic Circle. Edifices such as the remaining 28 Viking Stave Churches, which are 800–900 years old, and the ancient Viking ships preserved in sea mud or at burial sites, underline the deeply rooted wood construction traditions. Most buildings were, however, subject to neglected maintenance and repair and lost to the deterioration processes associated with a generally humid climate. However, since wood burns, structures and even whole villages and towns were also lost through the centuries in devastating conflagrations [5].

The building fire frequency is highest during winter time in Norway, partly due to the use of open flames, for example, candles, during the Advent, Christmas and New Year celebrations, that is, late November to early January. However, the use of fireplaces, wood burners and candles continue during January and February in the dark and cold winter. Electricity is the most common heating mode. During cold periods with excessive electricity consumption, old electrical wiring is known to start fires due to overheating [6]. Chimney fires also result in increased fire frequency during winter time. Low ambient temperatures, with low dew point, have also been acknowledged as a factor contributing to the high winter time fire frequency, as explained by Pirsko and Fons [7], based on selected low temperature areas in the USA. Babrauskas [8] assumed that the connection between low dew point and high fire frequency would probably also be valid for modern buildings.

In Norway, the temperatures during the winter may drop to well below −30 °C inland. Along the west coast, wind from the west brings humid air from the Atlantic Ocean. Wind from the east brings adiabatically heated (but often subzero) dry air plunging down from the central Norwegian mountain plains. Windy weather often follows dry, cold periods. The conflagrations in Norway during the period from 1900 to the present day generally occurred during winter time. The most well-known is the Ålesund fire on 23 January 1904. In storm strength winds, 850 structures were lost, resulting in 10,000–12,000 homeless. One person died in this conflagration [9]. A fire in Bergen, during 15–16 January 1916, resulted in two fatalities, 380 lost structures, 2700 homeless and 1000 people unemployed [5]. On 7 April 1923, a conflagration in Hemnesberget, just south of the Arctic Circle, destroyed 250 buildings [5], making it the most severe conflagration in Norway the last 100 years. In recent years, the 18–19 January 2014, fire raging through Lærdalsøyri destroyed 40 buildings, and ten days later, the Flatanger fire, just 100 km south of the Arctic Circle, destroyed 61 buildings, demonstrating that large winter fires in Norway are not just events of the past [10]. The fire spread mechanism in these two recent fires was identified as spotting ignition [11]. In combination with very strong winds, the severity of these fires may be explained by climatic conditions associated with subzero dry air and drying of indoor wooden materials [6,9]. These fires clearly demonstrate that conflagration risk should not be underestimated by the modern society.

Single structure fires may also be partly explained by climate conditions. The devastating fire at Résidence du Havre nursing home, L’Isle-Verte, QC, Canada, 23 January 2014, resulted in 32 fatalities and 15 injured [12], serving as an example. Adiabatically heated, dry, and still very cold, air further heated to indoor conditions resulted in a dry wooden structure very susceptible to fast fire development. This left the firefighters with very limited possibilities for fire intervention [13]. Official investigations of the mentioned three 2014 fires did, however, not mention the dry state of indoor combustibles as a risk factor [10,12]. The increased fire risk associated with very dry indoor wood apparently does not seem to be recognized.

Worldwide, recent major Wildland–Urban Interface (WUI) fires and large urban fires, for example, after earthquakes, have resulted in increased attention to large scale fires. An international effort to identify similarities in fire development and fire spread and suggest a roadmap for future research to mitigate such fire risks has recently been initiated [14,15]. These WUI fires have normally been observed in warmer and drier climate zones. However, the very dry 2018 spring and summer, with many forest fires, put drying of potential wildland fire fuel on the agenda in Norway.

There is a strong relationship between fire service response time and the outcome of fires [16,17]. In general, the faster the firefighters get involved in active firefighting, the better the outcome of their efforts will be. Given a constant response time, a comparably faster fire development will result in a more developed fire when the emergency responders arrive on scene. Studies of vertical flame spread in wooden corners revealed a much faster fire spread for low moisture content wood than more humid wood [18]. It has also been demonstrated that the time needed for a 2.5 kW fire to give flashover in ¼ scale wooden (pine) test rooms was highly dependent on the wood fuel moisture content (FMC) [19]. For 9 wt % (weight percent) FMC, corresponding to about 50% relative humidity (RH) [20], the time to flashover was just over 8 min. For 4.5 wt % FMC, corresponding to 20% RH, the time to flashover was reduced to 4 min. Taking into consideration turnout time and time to get water on fire (WOF), the available transport time from a fire station to get WOF before flashover is dramatically reduced during dry conditions. Shorter available travel times significantly reduce the fire station coverage area before a fire is threatening to the inhabitants as well as to the neighbor structures. During windy conditions, wooden home flashovers may result in longer transport distances for glowing embers and firebrands igniting distant structures. This makes firefighting efforts extremely challenging [9,21,22].

A future forecasting system for dry wood materials fire risk based on recorded weather parameters from professional weather stations combined with weather forecasts may be a valuable approach for contingency planning [23] and resource allocation [24]. Since it is the dry indoor climate that eventually leads to dry indoor wood materials, the indoor relative humidity needs to be monitored or modeled in such an approach. Indoor RH is usually recorded and modeled for a number of other reasons than fire risk. Too high humidity levels may potentially lead to deterioration of wooden constructions [25,26]. It is central regarding energy optimization [27,28] and represents an important air quality parameter [29,30]. Indoor temperature and humidity stability was studied by Kalamees et al. [31] for detached homes with varying ventilation systems and building fabrics in Finland. By continuously recording temperature and relative humidity in bedrooms and living rooms, they concluded that ventilation had more effect on the indoor climate than the types of buildings and the properties of the building fabric. Textiles and furniture were also recognized as playing a significant role in indoor relative humidity dampening, together with window airing, etc. Although it is influenced by several parameters, the indoor relative humidity is a key to assessing moisture content of indoor wood-based potential fire fuel.

The purpose of the present study was to develop a cold climate fire risk model for the first home involved in fire based on modeled indoor relative humidity and transient drying of wooden wall panels. The research focuses on inhabited (heated) wooden homes in densely built towns areas in Norwegian regions historically associated with severe winter time conflagrations. The modeled indoor relative humidity, based on ambient weather data, is compared with recorded indoor relative humidity for selected homes. The modeled indoor wood panel moisture content is considered as an independent building fire risk contributor due to its influence on the estimated time to flashover. The article is organized in parts describing the theoretical background, modeling methodology and model input parameters, the model results compared to recorded indoor relative humidity, and the strengths and weaknesses of the model in predicting single home fire risk and conflagration risk. In retrospect, the model was used to evaluate whether any days during the winter 2016 were associated with high conflagration risk in the area studied. The potential of using the model in combination with weather forecasts for predicting individual home conflagration risk a few days into the future for possible risk based emergency responses is also discussed. No similar previous work has been identified in the research literature.

## 2. Theory and Model Description

### 2.1. Gas Phase Water Vapor Concentrations

The equilibrium (saturated) water vapor pressure is nearly an exponential function of temperature, *T*_c_ (°C), and may be calculated by [32]

(1) $$P_{w,sat}=610.78\cdot e^{\left(\frac{17.2694\cdot T_{c}}{T_{c}+238.3}\right)}(\mathrm{Pa}),$$

The saturation concentration of water in the gas phase at a given temperature *T* (K), i.e., *T*_c_ + 273.15 K, is obtained by

(2) $$C_{w,sat}=\frac{P_{w,sat}\cdot M_{w}}{R\cdot T}\;(\mathrm{kg}/m^{3}),$$

where $M_{w}$ (0.01802 kg/mol) is the water molecular mass and R (8.314 J/K∙mol) is the gas constant. The relative humidity represents the actual concentration of water in the air relative to the saturation concentration, that is, RH = $P_{w}$/$P_{w,\mathrm{sat}}$ = $C_{w}$/$C_{w,\mathrm{sat}}$.

**Figure 1:**
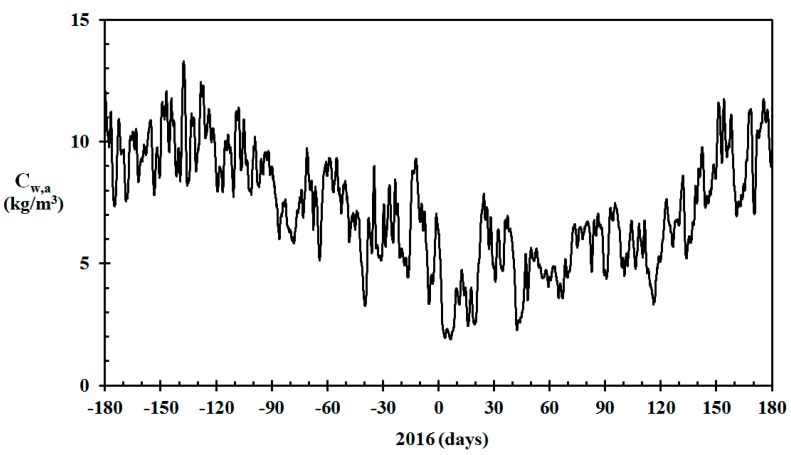
Ambient water vapor concentration (24 h average) for Haugesund Airport, July 2015 to June 2016. (Temperature and relative humidity data from www.eklima.no).

In the Western Norway coastal areas, the ambient water vapor concentration varies considerably during the year. Ambient temperature and relative humidity for Norwegian meteorological stations may be retrieved for free from www.eklima.no. The ambient water vapor concentration based on data recorded at the Haugesund Airport meteorological station (N 59.3448, E 5.2107), that is, $C_{w}\;$= RH·$C_{w,\mathrm{sat}}$, from 1 July to 30 July 2015, is shown in Figure 1. It is seen that the water vapor concentration was especially low during periods in the winter.

When low temperature ambient air at, for example, −10°C is ventilated into, or penetrates into, an inhabited building, it is heated to a temperature associated with a significantly higher water vapor saturation concentration. Additionally, the entrained air expands according to the ideal gas law and the water concentration of the entrained air becomes

(3) $$C_{\mathrm{in}}=C_{\mathrm{out}}\frac{T_{\mathrm{out}}}{T_{\mathrm{in}}}\;(\mathrm{kg}/m^{3}),$$

where $T_{\mathrm{out}}$ (K) and $T_{\mathrm{in}}$ (K) are the outdoor and indoor temperatures, respectively. The relative humidity of the entrained air is given by

(4) $${\mathrm{RH}}_{\mathrm{in}}={\mathrm{RH}}_{\mathrm{out}}\left(\frac{P_{\mathrm{sat}}\left(T_{\mathrm{out}}\right)}{P_{\mathrm{sat}}\left(T_{\mathrm{in}}\right)}\right)\left(\frac{T_{\mathrm{out}}}{T_{\mathrm{in}}}\right)$$

Inside the building, the entrained air then mixes with the indoor air. There are usually internal sources of moisture supply, ${\dot{m}}_{s}$ (kg/s), for example, individuals breathing and perspiring, cooking, pets and plants, etc. releasing humidity. The number of occupants present in any given home may vary from families where most of the members stay indoors, to single dwellers hardly spending any time there. The occupancy may also vary greatly within the course of each day and between work days and weekends. Furthermore, when considering a ground floor living room, people may sleep in an upstairs floor. The stack effect during cold weather then results in a minimum of humidity transport down to the living room. Much work has been done regarding average moisture supply in homes [31,33,34] and it should be noted that an average building or an average user probably does not exist. Assuming a moisture supply on the order of 1 kg/day in the living room seems reasonable, unless other information indicates otherwise.

The number of air changes per hour (ACH) is normally used as a measure of the air ventilation rate. The relative air dilution due to a known air exchange rate (ACH) may be expressed as

(5) β=1−exp(−ACH·Δt/3600 s)

Building codes have changed much over the years, and may significantly influence the building envelope air tightness [28]. The build quality may also differ. The codes and regulations have gradually been improved to ensure proper ventilation to prevent fungi development, rot, etc., as well as to ensure proper indoor air quality. A typical requirement for modern homes with balanced ventilation is 0.5 ACH. In calm weather, and under moderate indoor-to-outdoor temperature differences, a significantly lower air change rate may be observed in naturally ventilated homes. In such buildings, the air change rate generally increases with temperature differences giving stack effects and/or wind speed. Window airing may significantly increase the average ventilation rate. For older wooden homes, a proper guess may be to assume half the number of ACH compared to balanced ventilated buildings, that is, 0.25 ACH.

### 2.2. Wood Equilibrium Moisture Content (EMC)

**Figure 2:**
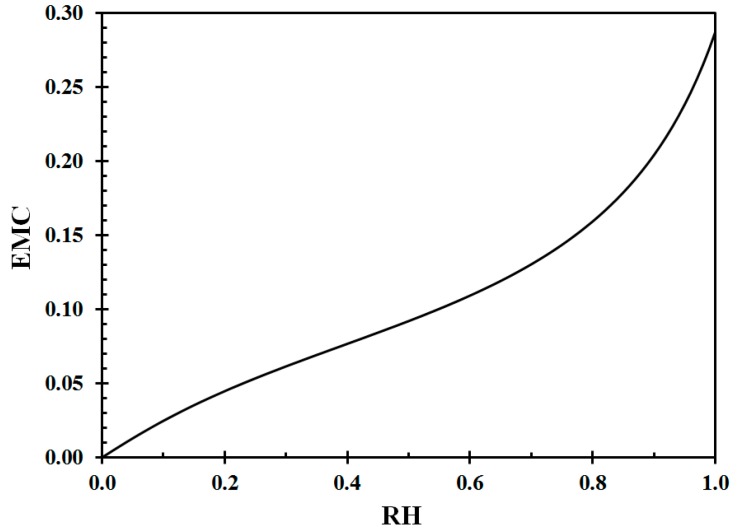
Equilibrium moisture content (EMC) at 22 °C as a function of relative humidity (RH). Data from Equation (6) [20].

**Figure 3:**
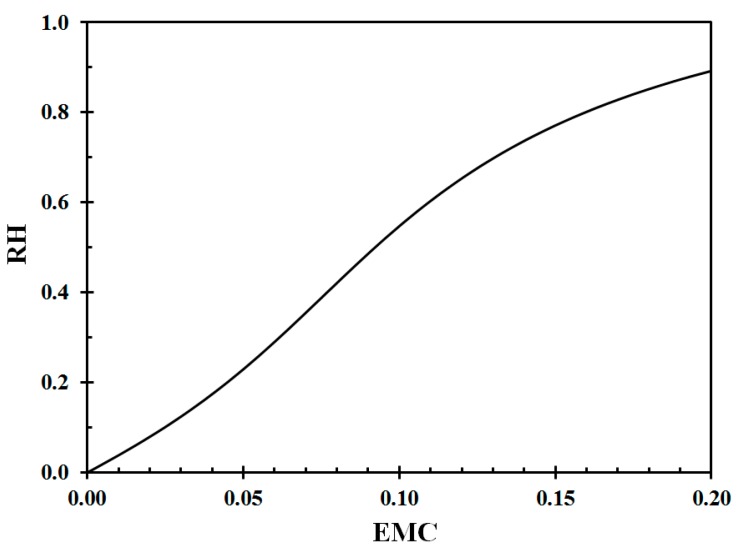
Relative humidity (RH) as a function of equilibrium moisture content (EMC) at 22 °C. Data from Equation (6) [20].

Dependent on the previous exposure, cellulose based materials, such as clothing, furniture, wooden wall panels, floors and ceilings, may release or adsorb humidity from the indoor air. Wood and other cellulose based materials display a very complicated molecular structure with free hydroxyl groups. This results in hysteresis when exposed to cycles of humid and dry air [35,36]. However, given long time, that is, *t* → ∞, the corresponding wood EMC may typically be calculated by [20]

(6) $$\mathrm{EMC}=\frac{1800}{W}\left\{\frac{Kh}{1-Kh}+\frac{K_{1}Kh+2K_{1}K_{2}K^{2}h^{2}}{1+K_{1}Kh+K_{1}K_{2}K^{2}h^{2}}\right\}$$

where

$$W=349+1.29T+0.0135T_{c}^{2}$$

$$\;K=0.805+0.000736T_{c}-0.00000273T_{c}^{2}$$

$$\;K_{1}=6.27-0.00938T_{c}-0.000303T_{c}^{2}$$

$$\;K_{2}=1.91+0.0407T_{c}-0.000293T_{c}^{2}$$

where h is the relative humidity fraction (%/100). The EMC for wood, according to Equation (6), at 22 °C is shown in Figure 2. The inverse curve is presented in Figure 3.

For modeling purposes, in the present work, fourth order polynomials were fitted to the data presented in Figure 2 and Figure 3 for the relative humidity range of interest in the study, that is, 10% RH to 80% RH, respectively, giving

(7) $$\mathrm{FMC}=0.0017+0.2524\cdot \mathrm{RH}-0.1986\cdot {\mathrm{RH}}^{2}+0.0279\cdot {\mathrm{RH}}^{3}+0.167\cdot {\mathrm{RH}}^{4}$$

(8) $$\mathrm{RH}=0.0698+1.258\cdot \mathrm{FMC}-125.35\cdot {\mathrm{FMC}}^{2}+809.43\cdot {\mathrm{FMC}}^{3}+1583.8\cdot {\mathrm{FMC}}^{4}\;$$

The regression coefficients for Equations (7) and (8) were very close to unity. For simplicity, assuming that the hysteresis does not dominate the wood sorption processes, Equations (7) and (8) can then be used to model the transport of humidity between the wooden surfaces and the indoor air in direct contact with the wood surface.

### 2.3. Transport of Humidity in Wood

Fires in wooden homes often involve the walls during fire development towards flashover [37]. Tests, for example, the ISO 9705 Room Corner Test [38], are therefore arranged to analyze ignition and involvement of the wall materials under given floor level heat release rates. In order to test the modeling of humidity transport in representative wood products, wall panels were selected as a representative fuel. For simplicity, it may be assumed that the diffusion coefficient is independent of the wood water concentration for the range of interest in the present work, that is, 20% RH to 50% RH at a constant indoor temperature, for example, 22 °C. The diffusion of humidity in the wood may then be described by Ficks law:

(9) $$\frac{\partial C}{\partial t}=D_{w,s}\cdot \nabla^{2}C$$

where C (kg/m^3^) is the wood water concentration and $D_{W}$ (m^2^/s) is the water diffusion coefficient in the wood. The convective transport of humidity from the wood surface to the indoor air may be expressed by

(10) $${\dot{m}}_{\mathrm{wall}}=A\cdot \frac{D_{w,a}}{\delta }\cdot \Delta C$$

where A (m^2^) is the exposed surface area of the wood panel, $D_{w,a}$ (m^2^/s) is the diffusion coefficient of water vapor in air and δ (m) is the boundary layer thickness. ΔC (kg/m^3^) represents the concentration difference between the wood panel surface air water concentration based on Equation (8) and the indoor bulk air water concentration. The parameter $D_{w,a}/\delta$ is equivalent to the convective heat transfer coefficient, h=k/δ (W/m^2^ K), in heat transfer, that is, where k (W/m K) is the air thermal conductivity. When studying diffusion through paper, Mortensen [39] demonstrated that under normal conditions the air resistance is of less importance than two layers of 190 g/m^2^ paper (about 0.4 mm total thickness). It is, therefore, not expected that the modeling in the present work would be very dependent on the boundary level thickness, δ, when selected within reasonable limits. Utilizing the heat transfer equivalent, and assuming a comparably low heat transfer coefficient of 2.6 W/m^2^·K and an air thermal conductivity 0.026 W/m·K, the boundary layer thickness turns out to be in the order of 0.01 m. This boundary layer thickness, that is, δ = 0.01 m, was therefore used in the present work for the wall to air water vapor exchange.

In order to model the transport of moisture within the wood panels, the panels were sliced in *N* slabs of thickness Δx (m). For the wood surface layer *N* = 1, Equation (9) may be discretized as

(11) $$C_{1\left(t+\Delta t\right)}=C_{1\left(t\right)}+\frac{\Delta t}{A\cdot \Delta x}\left\{\frac{D_{w,a}}{\partial }\left({\mathrm{RH}}_{\left(t\right)}-{\mathrm{RH}}_{1\left(t\right)}\right)C_{\mathrm{sat},22{}^{\circ}C}+\frac{D_{w,s}}{\Delta x}\left(C_{2\left(t\right)}-C_{1\left(t\right)}\right)\right\}\;\;(\mathrm{kg}/m^{3})$$

where ${\mathrm{RH}}_{1\left(t\right)}$ is calculated by Equation (8) based on $C_{1\left(t\right)}$. For the bulk layers from *n* = 2 to *n* = *N* − 1, the new concentration may be calculated by

(12) $$C_{n\left(t+\Delta t\right)}=C_{n\left(t\right)}+Fo\cdot \left(C_{n-1\left(t\right)}-2\cdot C_{n\left(t\right)}+C_{n+1\left(t\right)}\right)\;(\mathrm{kg}/m^{3})\;$$

where

(13) $$Fo=\;\frac{D_{w,s}\cdot \Delta t}{\Delta x^{2}}$$

For external walls, a moisture diffusion barrier is introduced in buildings in cold climates to prevent humidity transport to lower temperature areas, condensation and rot formation [40]. For layer *N*, it was therefore assumed that there was no exchange of water at the rear surface, that is, only moisture exchange with the inside layer (number *N −* 1):

(14) $$C_{N\left(t+\Delta t\right)}=C_{N\left(t\right)}+\;Fo\cdot \left(C_{n-1\left(t\right)}-C_{n\left(t\right)}\right)\;\;(\mathrm{kg}/m^{3})$$

It should be noted that the integration time interval, Δt, must comply with Fo < 0.5 to ensure numerical stability.

According to Baronas et al. [41], typical diffusion coefficients of water in wood are in the range 1–5 × 10^−10^ m^2^/s. A value in the center of this range, that is, 3.0 × 10^−10^ m^2^/s, was therefore taken as a representative value for $D_{w,s}$ in the current study. Norwegian indoor wood lining panels are typically 10 or 12 mm thick. Since there are also usually wooden floor panels of at least 12 mm thickness in these buildings, a thickness of 12 mm was therefore assumed as representative for the modeling. Paint and lacquer, linoleum cover on the floors, etc., may act like surface layers limiting the moisture exchange between the indoor air and the internal wood surfaces. This may be handled by assuming that only a certain fraction, ξ, of the indoor wood surface area is active in the moisture exchange.

### 2.4. The Overall Numerical Model

In order to model the indoor air relative humidity, knowledge about air change rate, indoor volume, water diffusion coefficients in air and in wood, the boundary layer distance, moisture supply, and the surface area and thickness of the involved wood panels, is required. Assuming that these values are known, a mass balance for water in the indoor air volume may be established:

(15) $$V\frac{dC}{dt}={\dot{m}}_{\mathrm{AC}}+{\dot{m}}_{\mathrm{wall}}+{\dot{m}}_{\mathrm{supply}}\;(\mathrm{kg}/s)$$

The initial conditions can simply be taken as the values from a representative day a few days prior to the period of interest in order for the indoor water concentration to adjust to reasonable values. Then, modeling forward in time can be done based on recorded ambient temperature and relative humidity.

## 3. Results

### 3.1. Testing the Model for a 120 Year-Old Wooden Home

**Figure 4:**
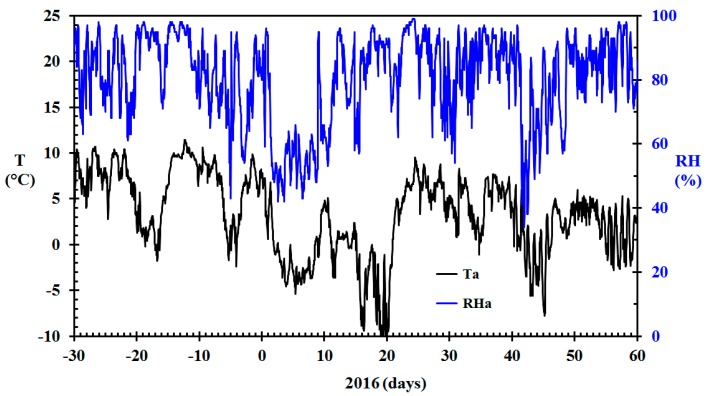
Ambient temperature (*T*) and relative humidity (RH) for Haugesund Airport in the period December 2015 through February 2016. (Data from www.eklima.no).

**Figure 5:**
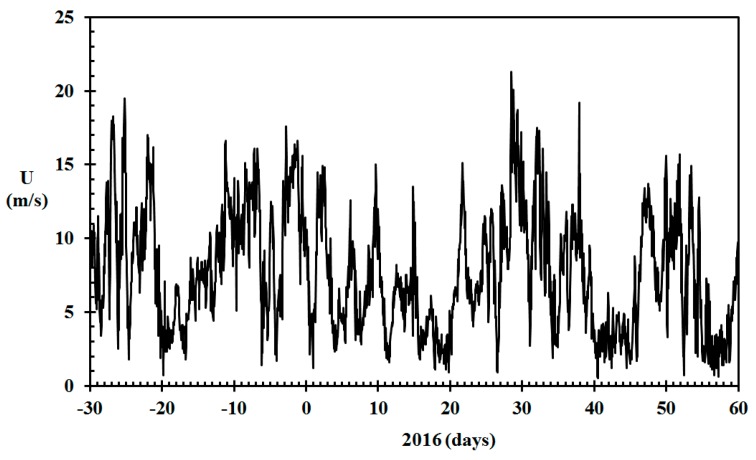
Wind speed, *U* (m/s), for Haugesund Airport in the period December 2015 through February 2016. (Data from www.eklima.no).

Future fire risk prediction could rely on the pre-existing network of professional weather stations. This would eliminate any requirement for dedicated weather stations, in need of calibration and maintenance, in the areas of interest. Thus, the basis for the modeling of indoor relative humidity in the present work was temperature and relative humidity data collected for free at the closest professionally maintained meteorological station (www.eklima.no). It has previously been documented that these stations give results in compliance with outdoor temperature and humidity sensor recorded in the areas of interest [6]. The temperature and relative humidity recorded by the Haugesund airport meteorological station for December 2015 through February 2016 are presented in Figure 4. The recorded wind speed for the same period is presented in Figure 5.

**Figure 6:**
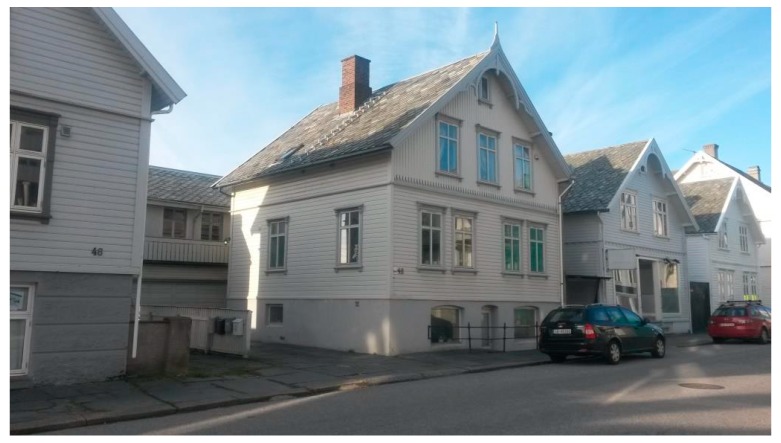
The home built in 1910.

It was decided to test the model for a wooden home built in 1910 in Haugesund town center, Norway (N 59.4083, E 5.2750). The home, as shown in Figure 6, is a timber frame construction typical for that time period [40]. The internal walls in contact with the indoor air consist of 10–12 mm wood linings (pine or fir) on a vapor air barrier. Then, there is a layer of thermal insulation which in homes of this age were solid wood, 2–3″ thick. Following this, there is a wind barrier, a ventilated space and finally, the external rain protective wood cladding. The home studied has an indoor wood surface contact area of 100 m^2^.

**Figure 7:**
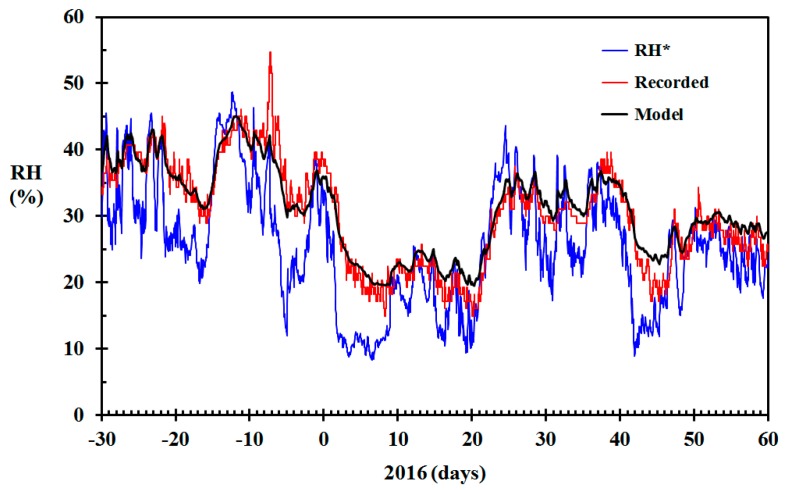
Modeled relative humidity (RH) (black) based on the data from Figure 4, recorded RH in the living room (red) and RH* based on infinite air change rate (blue) December 2015 through February 2016 for the home built in 1910.

The modeled indoor RH (black) and the indoor RH based on Equation (4) (blue) are shown in Figure 7. The figure also shows the indoor RH (red) in the living room recorded using Netatmo sensors calibrated to within ±3% RH, as outlined in [42]. Basing the indoor RH at 22 °C on Equation (4) versus the recorded indoor RH resulted in a root mean square (RMS) error of 7.4% RH. This high RMS error demonstrates that ignoring the humidity modelling and directly applying Equation (4) gives erroneous results when dynamic changes in ambient temperature and relative humidity is experienced. Comparing the modeled RH (black) to the recorded RH (red), the RMS error was reduced to 2.5% RH. The largest positive difference was 6.7% RH. One conspicuous peak in the recorded RH, as seen in Figure 7, gave a largest negative difference of 14.3% RH.

All the relative humidity sensors were calibrated against inorganic salt atmospheres, as described in [42] so that their recorded values were well within 3% RH. Any deviations larger than ±3% RH between the modeled and recorded RH must, therefore, be due to limitations in the model. In Figure 7 it is, however, seen that the modeled indoor RH agrees quite well with the recorded values during the period December through February. The conspicuous peak in recorded indoor RH on 24 December, causing the −14.3% RH error, is probably due to the Christmas celebration. There is, however, a tendency for the modeled RH to systematically deviate from the recorded RH during the driest indoor periods. In this geographic region, such dry periods during winter time are generally associated with low outdoor temperatures. The deviation may, therefore, simply be due to a larger air change rate during these troughs. Assuming that the stack effect is responsible for the inflow of ambient air, the inflow rate is, according to the Bernoulli principle, proportional to

(16) $${\dot{m}}_{\mathrm{air}}\propto \varrho_{a}\;\sqrt{\left(\varrho_{a}-\varrho_{\mathrm{in}}\right)/\varrho_{a}}\propto \sqrt{\left(1/T_{a}-1/T_{\mathrm{in}}\right)/T_{a}}\;(\mathrm{kg}/s)\;$$

where $\varrho_{a}$ (kg/m^3^) is the density of ambient air, $\varrho_{\mathrm{in}}$ (kg/m^3^) is the density of indoor air, $T_{a}$ (K) is the ambient temperature and $T_{\mathrm{in}}$ (K) is the indoor temperature. For simplicity, the air change rate was therefore adjusted as shown in Equation (16), with a constant, γ, to match proper ventilation rates, that is,

(17) $$\mathrm{ACH}=\gamma \cdot \sqrt{\left(1/T_{a}-1/T_{\mathrm{in}}\right)/T_{a}}\;(1/h)\;$$

**Figure 8:**
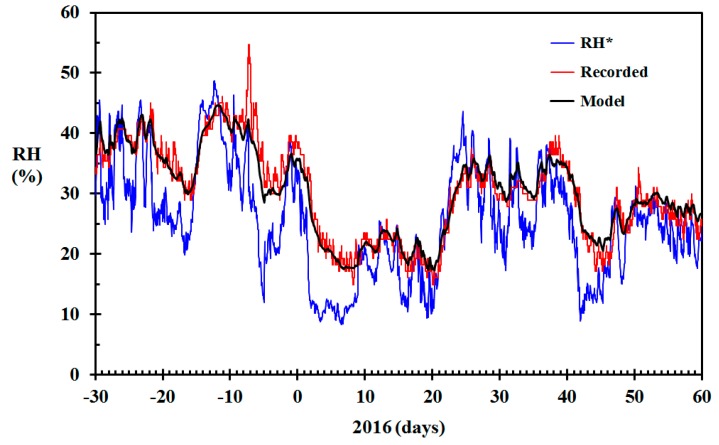
Modeled relative humidity (RH) (black) based on the data from Figure 4 and air change rate based on Equation (16) (γ = 300 h^−1^), RH recorded in the living room (red) and RH* based on infinite air change rate (blue) December 2015 through February 2016 for the home built in 1910.

For an outdoor temperature of 6 °C, selecting γ = 300 h^−1^ gives ACH = 0.250 h^−1^. For −5 °C it gives ACH = 0.338 h^−1^. Using this value for γ in Equation (17) gave modeled indoor RH closer to the recorded values, as seen in Figure 8, especially for the cold, that is, dry indoor conditions, periods. The root mean square (RMS) error was then improved from 2.5% RH to 2.1% RH. The largest positive difference was improved from 6.7% RH to 4.8% RH. The largest negative difference was not altered, since this deviation was related to the substantial peak in the recorded indoor RH.

It could be argued that the wind speed should also be taken into account. It is, however, difficult to know the wind speed within a densely built town environment, and it will certainly be much lower than at the Haugesund airport, which is located in open terrain along the North Sea at N 59.3448, E 5.2107. Larger modern buildings in the town area partly shield the wind impact on the lower old wooden homes. The influence of wind may also depend on the wind direction [43]. Since low temperatures in this geographic area are also generally associated with low wind speed, as seen in Figure 4 and Figure 5, it was not attempted to adjust for the wind speed recorded at the Haugesund Airport.

Testing the modelling with a boundary layer distance, δ, of 0.02 m or 0.005 m, that is, double or half the suggested reasonable value, did not change the results. It was therefore decided that δ = 0.01 m was a sound estimate. Furthermore, using a wood moisture diffusion coefficient, $D_{w,s}$, of 2 × 10^−10^ m^2^/s and 4 × 10^−10^ m^2^/s, did not change the results significantly. It was, therefore, concluded that 3 × 10^−10^ m^2^/s was a fair estimate for the wood moisture diffusion coefficient.

### 3.2. Testing the Model for a 100 Year-Old Wooden Home

**Figure 9:**
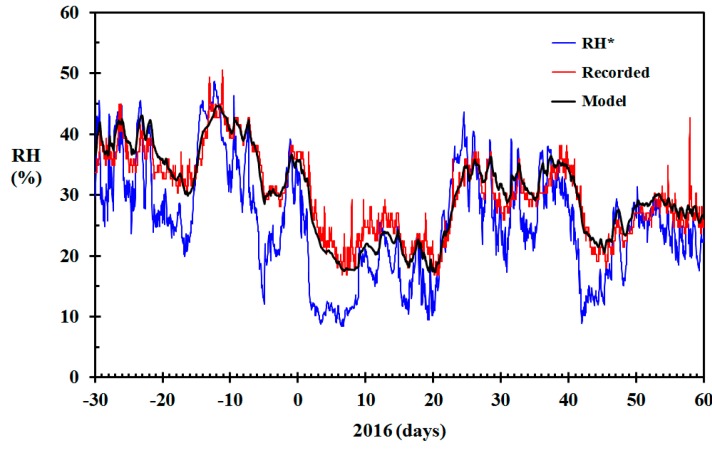
Modeled relative humidity (RH) (black) based on the data from Figure 4 (γ = 300 h^−1^), RH recorded in the living room (red) and RH* based on infinite air change rate (blue) December 2015 through February 2016 for the home built in 1917.

It was decided to test the model for predicting the indoor relative humidity of a wooden home built in 1917 and located in Haugesund, Norway, at N 59.4166 and E 5.2686, based on the meteorological observations presented in Figure 3. This home was quite similar to the home built seven years earlier, with a footprint of about 80 m^2^ and an indoor wood surface contact area of approximately 100 m^2^. The air change rate adjusted for stack effect, as suggested in Equation (17), with γ = 300 h^−1^, was therefore used for the modeling. The results are shown in Figure 9, including the recorded indoor RH values. The RMS error of the modeled RH versus the recorded RH was 2.2% RH.

It can be seen that the modeled results generally agree well with the recorded indoor RH. Around 10 January, the modeled indoor RH was lower than the recorded values. The Netatmo indoor units also record the CO_2_ concentration, though with a quite unprecise self-calibration. Nevertheless, during this period, the CO_2_ concentrations recorded by the Netatmo indoor unit were quite high, indicating either high levels of human activity or low ventilation rates, explaining the observed deviation. In mid-February, the modeled RH gave some higher relative humidity than the recoded values. According to the CO_2_ concentrations, there seemed to be no human activity at day 43, 44, 48, 49 and 50, and low human activity day 45, 46 and 47. It was later confirmed that the inhabitants were away from day 42 to 51. This may at least partly explain the deviations between modeled and recorded indoor RH during mid-February. The model was, however, not designed to take such variations in human activity into consideration. For this particular home, the average, maximum positive and largest negative deviation of the modeled indoor RH compared to the recorded indoor RH was −0.64% RH, 5.4% RH and −18.6% RH, respectively. The largest negative deviation was associated with the conspicuous peak in RH recorded at the end of the period. This peak was most likely real, that is, due to high human activity, rather than a spurious recording. The root means square error for the modeled versus the recorded indoor RH during the period shown in Figure 9 was 2.2% RH.

### 3.3. Testing the Model for a 55 Year-Old Wooden Home

**Figure 10:**
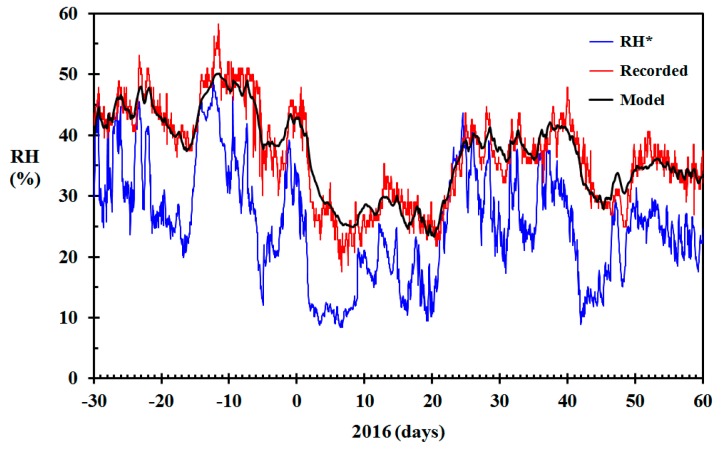
Modeled relative humidity (RH) (black) based on the data from Figure 4 (γ = 200 h^−1^), RH recorded in the living room (red) and RH* based on infinite air change rate (blue) December 2015 through February 2016 for the home built in 1963.

A 55 year-old wooden home close to Haugesund, located in quite an open landscape at N 59.3717, E 5.3030, was also selected for testing the model. This home is also a timber frame construction, but differs from the two older homes in that the wall thermal insulation consists of mineral wool, quite typical for this time period [40]. This home was much larger than the older town homes, with a footprint of about 150 m^2^. Using γ = 300 h^−1^ in Equation (17) did not give a good fit when modeling the indoor relative humidity. Using γ = 200 h^−1^ did, however, give a fair fit, as seen in Figure 10. Selecting a lower γ value in this case may be justified based on the larger volume to wall area ratio. The building envelope may also have been less permeable compared to the two homes from the early 1900s.

For this particular home, larger deviations were experienced between the modeling and the indoor results, and for some days the indoor relative humidity was lower than expected, for example, during the first days of January. It is reasonable that this detached home, located in a rural open landscape, was to a greater extent influenced by wind than the shielded homes in the town center. Though the model deviates to some extent during the driest period, that is, in early January, the model still provides information about quite dry indoor conditions. The root mean square error for the modeled versus the recorded indoor RH during the period shown in Figure 10 was 2.5% RH.

### 3.4. Testing the Model for a Balanced Ventilation Modern Wooden Home

**Figure 11:**
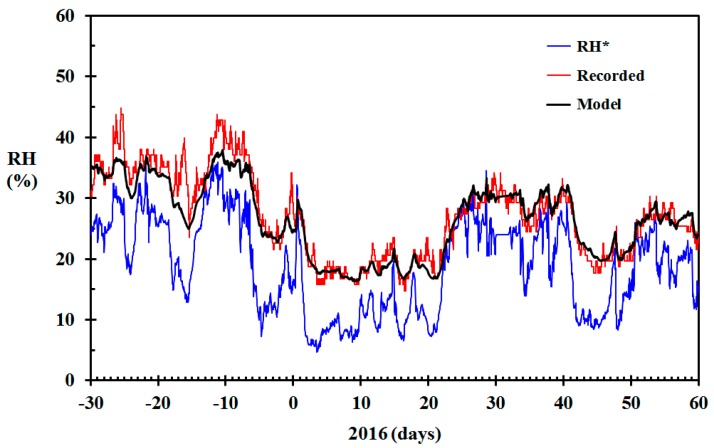
Modeled relative humidity (black) based on the data from the Lærdal meteorological station (eklima.no) and 0.5 ACH, RH recorded in the living room (red) and RH* based on infinite air change rate (blue) December 2015 through February 2016 for the balanced ventilation home.

New wooden homes in Norway are generally built with balanced ventilation for better indoor air quality control and efficient heat exchange between the exhaust air and the supplied air. They are also timber frame constructions, but have even thicker thermal insulation than the homes constructed in the previous years in order to minimize heat losses. They are quite impermeable and operate under a slight negative pressure to prevent humid air from the indoor environment to penetrate out through the building envelope. For modeling the indoor RH of a modern home with balanced ventilation, a home in Lærdal valley at N 61.1003, E 7.4900, was selected. This home is quite typical for modern wooden homes in Norway, with wooden parquet flooring and gypsum plaster board wall linings. The balanced ventilation generally overcomes the stack effects, that is, the 0.5 ACH forced ventilation dominates the ventilation. This constant value was therefore used in the modeling. The result is shown in Figure 11, including the recorded indoor RH values. The ambient air data were retrieved from the Lærdal meteorological station (www.eklima.no). It should be noted that the air in Lærdal valley is generally dry due to being adiabatically heated [9], as seen by the low RH* in Figure 11. The root mean square error for the modeled versus the recorded indoor RH during the period shown in Figure 10 was 2.4% RH.

The deviation between the modeled and recorded indoor RH 10 days before New Year is most likely due to humidity released from an open river 80 m north of the home. This was confirmed by higher outdoor moisture content detected by an outdoor sensor compared to the meteorological station for that period. The deviation between recorded and modeled RH in early December coexisted with high indoor CO_2_ concentrations, indicating either high levels of human activity or reduced air ventilation.

### 3.5. Indoor Wood Panels Moisture Content

**Figure 12:**
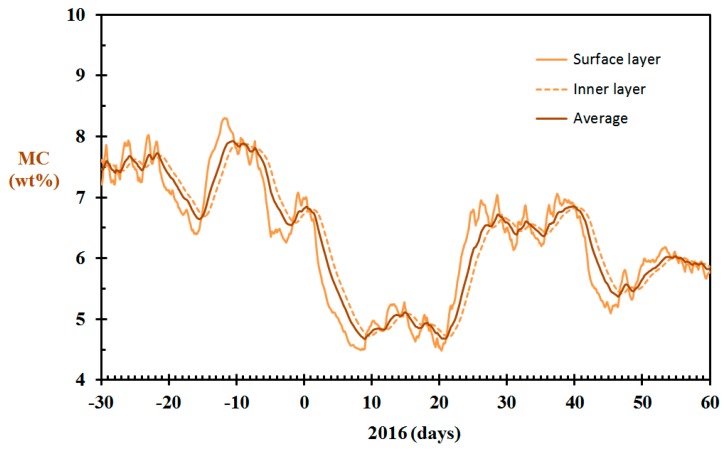
Wall panel moisture content (MC) for the home built in 1917, December 2015 through February 2016, modeled based on the data from Figure 4 and γ = 300 h^−1^.

The modeled wall panel moisture content for the home built in 1910, using Equation (17) and γ = 300 h^−1^, is shown in Figure 12. For considering the fire risk associated with dry indoor wood products, one may argue whether the surface layer moisture content or the average fuel moisture content (FMC) is most representative. Both are presented in Figure 12, together with the inner wood panel layer moisture content. It is clearly seen that the wood panels must have been very dry, and thereby susceptible to intense combustion if involved in fire during periods of the winter 2016.

### 3.6. A Possible Wooden Home Cold Climate Fire Risk Proxy

Based on studies of fire development in ¼ scale wooden compartments, Kraaijeveld et al. [19] found that the time to flashover was very dependent on the wood moisture content. Given a 2.5 kW start fire in the corner, and an open door, as in the ISO Room Corner Fire Test [38], the time to flashover for wood moisture content (WMC) in the range 0.04–0.13 was given by

(18) $$t_{\mathrm{FO}}=2.0\cdot \exp\left(16\cdot \mathrm{WMC}\right)\;(\mathrm{minutes})$$

**Figure 13:**
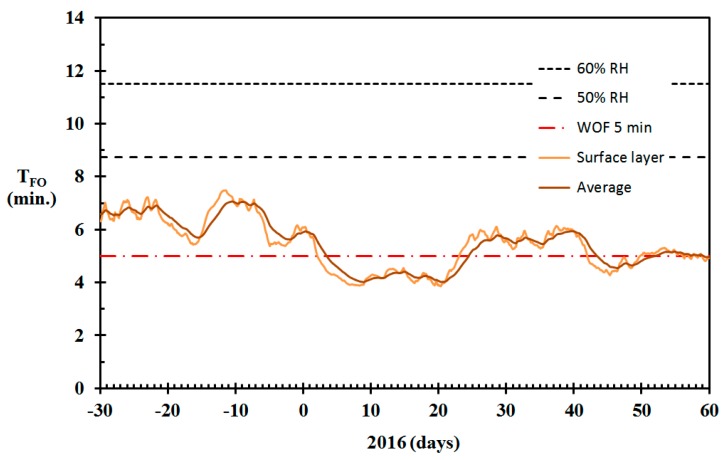
Estimated time to flashover (Equation (18)) for ¼ scale wooden compartments when in equilibrium at 60% relative humidity (RH), 50% RH and the moisture content calculated for the surface layer and the average wood panel moisture content in the period December 2015 through February 2016. (Wood moisture data from Figure 12.) The red dashed line represents 5 min to water on fire (WOF).

When the wood panel moisture content is known, as shown in Figure 12, it may be used for fire risk modeling exemplified by the estimated time to flashover. The calculated time to flashover based on the moisture content reported in Figure 12 and Equation (18) is shown in Figure 13. It should be noted that for standardized fire testing, it is usually required that hygroscopic products, for example, wood, should be equilibrated to 50% RH prior to the fire testing. In Western Norway, the indoor relative humidity may be well above 50% RH in the summer and fall seasons. The estimated time to flashover for wood in equilibrium with air at 50% RH and 60% RH are therefore also presented in Figure 13 as references to representative average conditions.

It is, however, well known that the time to flashover is dependent on the compartment involved [37]. This may be understood by analyzing typical characteristics for a compartment fire approaching flashover, which are:

- smoke layer temperatures approaching 500–600 °C,
- 20 kW/m^2^ heat flux to the floor level, and
- crumpled paper at the entrance of the compartment self-igniting.

**Figure 14:**
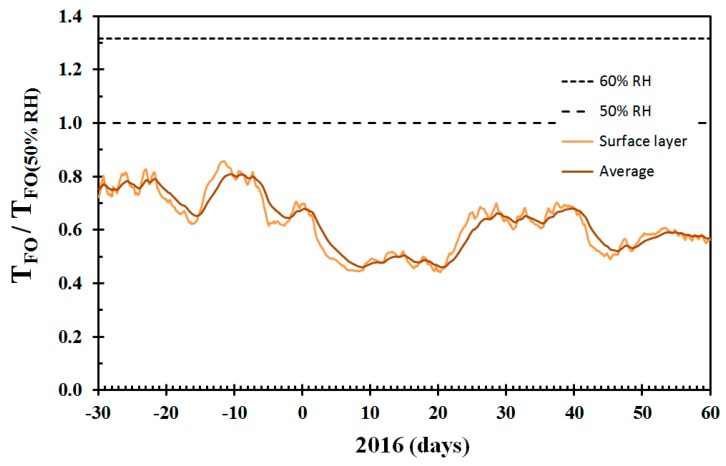
Time to flashover for the conditions presented in Figure 12 normalized by time to flashover at 50% RH (relative humidity) equilibrium conditions.

The time to reach flashover is dependent on the heat losses to the compartment boundaries, the ceiling height and the size of the compartment. A large smoke layer compartment contact surface would cool the smoke layer more than a smaller contact surface, etc. In order to take such issues into consideration, it may be better to present the time to flashover associated with dry wooden materials relative to a standard condition. Since fire testing usually requires the products to be tested to be acclimatized to 50% RH, this condition may then represent the standard condition. The data from Figure 13 normalized by the time to flashover at 50% RH is presented in this way in Figure 14. The dimensionless time to flashover may make it easier to recognize that flashover in a very dry building may be expected at a fraction of the time associated with normal (50% RH) conditions.

The relative time to flashover, as presented in Figure 14, may be taken as an indicator of the single dry wood structure fire risk. Compared to 50% RH conditions, the expected time to flashover was significantly shorter during winter time, especially for the period 8 January to 20 January 2016. Assuming that a structure may flashover in 10 min in normal conditions, it would be expected to reach flashover in 5 min in this period. This information can then be compared to the time required to detect an ongoing fire, warn the fire station, firefighter turnout time, driving time and the time needed to roll out fire hoses and get water on fire (WOF). It is quite clear that for some periods of the year, the fire would have developed beyond flashover, also for fires in structures within a few kilometers of the fire station. The fire fighters should then expect to arrive at the scene of a significantly more developed fire. In days with strong winds, the likelihood of fire spread to neighboring buildings would be very high. Such an analysis reveals that the transport time to reach a home before flashover is significantly reduced during dry indoor conditions [23].

Though the present work focusses on the first home involved in fire, some comments about the conflagration risk may be worthwhile. By comparing days of dry conditions (Figure 13 and Figure 14) and days of strong wind (Figure 5), it is possible to identify days of particularly high conflagration risk. By doing this, it may be concluded that in particular, January 9, 14 and 22, 2016, must have been associated with high conflagration risk in the densely built wooden town area of Haugesund. How high the conflagration risk was needs to be evaluated against both the wind speed and the relative humidity of the ambient air governing the likelihood of igniting neighbor structures. However, on, for example, 7 January, based on the dry wood and the weather forecast two days into the future, one could predict that 9 January would turn out to be a day of considerable conflagration risk. The same goes for predicting high conflagration risk a few days ahead of 14 and 22 January. To quantify the conflagration risk properly was, however, outside the scope of the present study.

### 3.7. Future Possibilities

The developed model may in the future be used for modeling single structure fire risk in representative inhabited single wooden buildings based on the weather forecast. If the conflagration risk had been understood prior to, for example, the Lærdalsøyri fire [9], mitigating measures could have been evaluated especially for the windy days. Manning the unmanned fire station during high risk days could have been considered. This would reduce the time to WOF considerably and increase the likelihood of containing the fire within the first building and probably prevent a conflagration. It would also be possible to extend the model for other densely built wooden towns in other cold climate regions, for example, in Japan, China, Chile, Canada, etc.

## 4. Discussion

In the present study, a numerical model for assessing the indoor relative humidity for wooden homes during winter time was developed and tested as a proxy for potential fast fire development. Ambient temperature and relative humidity recorded at meteorological stations were used as the external climatic boundary conditions. The model considers air exchange rate, indoor volume, sources of moisture supply and the transient effects of indoor wooden materials contributing to the water balance. The indoor wooden materials were represented by 12 mm thick wall panels and the model was tested for three selected wooden homes in the Haugesund area and one in Lærdal, Norway.

Inspired by the equivalent heat transfer coefficient, it was assumed that the modeling could be done satisfactorily with a constant air-to-wood humidity transfer coefficient. The humidity transfer from the wood panel surface to the indoor air was calculated based on a reasonable value for the heat transfer boundary layer, that is, 1 cm thickness. Testing for larger and smaller diffusion boundary layers did not influence the modeling. This is in agreement with the results obtained by Mortensen, et al. [39,44], where the boundary layer only became a limiting parameter when studying humidity transfer from thin layers of paper to indoor air. For simplicity, a constant moisture supply, dependent on the average number of inhabitants present in the studied homes, was an input to the model.

The modeled indoor RH was compared to the recorded indoor RH in the selected homes. The modeled indoor RH fit quite well with the recorded indoor RH for a water in wood diffusion coefficient of 3 × 10^−10^ m^2^/s, that is, in the middle of the range 1 × 10^−10^ m^2^/s to 5 × 10^−10^ m^2^/s recommended by Baronas et al. [41]. Testing with diffusion coefficients of 2 × 10^−10^ m^2^/s and 4 × 10^−10^ m^2^/s did not significantly alter the modeled indoor RH.

Assuming a constant air change rate of 0.25 h^−1^ gave a fair fit to the recorded indoor RH from December 2015 to February 2016 for 100+ years old homes in Haugesund. It was, however, observed that on the coldest days this ventilation rate resulted in the model giving slightly too high indoor RH. Introducing an air change rate proportional to the ambient and indoor temperatures Bernoulli stack effect air flow, with a proportionality constant of 300 h^−1^, produced a better fit, especially for the coldest days. The Bernoulli stack effect correction is therefore recommended for modeling indoor RH in old wooden buildings in cold climate, that is, buildings without mechanical ventilation.

For a larger wooden villa built in the 1960s, introducing a proportionality constant γ = 200 h^−1^ in the Bernoulli controlled ventilation rate, gave a good fit. The lower factor was justified based on two features, namely, the larger volume-to-surface ratio and the fact that this newer structure may have been a comparably less air-leaking construction. For a modern home with balanced ventilation, the correct constant air change rate, that is, 0.5 h^−1^, gave a good fit with the recorded indoor RH. Due to extensive use of plasterboard linings and less wooden surfaces exposed to the indoor air, the response time for adoption to drier conditions was faster for this home. This is also supported by other studies as plasterboard is known to be less hygroscopic than wood and thereby adapt faster to changing indoor RH conditions, that is, possessing a lower dampening effect [45,46].

The presented model did not consider the effects of hysteresis in the wood sorption processes. One reason for not considering hysteresis is that this effect is known to vary with the wood types involved [47]. Based on the generally good fit of the model with the recorded indoor RH, the modeling was evaluated as sufficiently accurate for estimating dry wood fire risk without further investigations into the complicated hysteresis mechanisms [48,49].

The humidity sensors were all calibrated to well within ±3% RH following the procedure suggested in [42]. Any deviation between the modeled and recorded values larger than this value was, therefore, a result of limitations in the modeling. The typical maximum positive and the largest negative deviation of the modeled versus recorded indoor RH was 5% RH and −14% RH, respectively. Large negative deviation for the studied homes was a result of high human activity. The root mean square error was within 3% RH.

It should, however, be noted that the indoor RH is highly dependent on the users of the buildings, their number, and presence as sources of humidity release, etc. Active window airing can also radically influence ventilation conditions and indoor RH [34]. In the present study, short-duration peaks in recoded indoor relative humidity levels well above the modeled results were observed for all the homes studied. These peaks were generally observed together with peaks in recorded CO_2_ concentration. The opposite was also the case. Lower recorded values of indoor relative humidity compared to the modeled results were found in cases where the indoor CO_2_ concentrations were close to ambient conditions, that is, close to 400 ppm CO_2_. The close connection between peaks and troughs in recorded RH compared to the modeling, and synchronized with CO_2_ concentrations, may indicate high and low (or non-existent) human activity, respectively. The model presented in the current study was not intended to reproduce such variations. The modeled results should, therefore, not be taken as the predicted indoor RH in a particular home, as that may temporarily vary considerably. It can, however, be regarded as an expected average for selected types of wooden homes based on the dynamically varying ambient temperature and relative humidity during winter time in Western Norway. For other countries, with different wooden home building traditions, building age distributions and air tightness, the factors used for modeling the air change rate may be quite different. It is, however, believed that the model as such can be adjusted to fit other wooden home building styles and work well also in other cold climate regions. It would also be interesting to test the model for historical wooden towns in other climatic regions in Norway. The Røros mountain village, a UNESCO World Heritage Site, and listed fishing communities in the Arctic areas could serve as sites for testing the model for heritage buildings in challenging climatic conditions.

During flashover, the heat release rate usually increases dramatically, and since the heat release rate is the single most important parameter in fire hazard [50], early flashover is a major challenge regarding firefighting efforts versus fire spread. Based on recorded time to flashover in ¼ scale wooden test compartments [19], the modeled wood panel’s fuel moisture content was used to estimate time to flashover as a contributor to dry wooden home fire risk. Though the modeled time to flashover is based on small scale measurements, it may serve as a fire risk model until full scale results for time to flashover as a function of wood moisture content are available. The presented methodology is thereby a first attempt to model wooden homes dry wood fire risk in cold weather. It should, however, be noted that if, for example, a large sofa with highly combustible upholstery represents the source of initial heat release, this object may exhibit a heat release rate sufficient to initiate flashover without involvement of any building materials. However, if this happens when wooden building materials are very dry, they will rapidly get involved and further accelerate the heat release rate. With respect to fire spread from one wooden structure to another, it is usually the building materials that contribute most, not the initially ignited object.

Modeled time to flashover may be compared with the time requirements for fire brigade interventions. According to Averill et al. [51] and Reglen and Scheller [52], this includes the time to verify a fire alarm and inform the alarm central (60 s), alarm-central processing time (60 s), firefighters turnout time (80 s, that is, for manned fire stations), transport time and time to get oriented, roll out fire hoses and start firefighting (40 s). The fire fighters’ turnout time, and the time from arrival at the scene of the fire to applying water on the fire, is known to vary during the day and throughout the year due to traffic and weather conditions [53]. If the response time for simplicity is assumed to be constant, the available travel time to reach burning wooden homes before flashover is significantly reduced when fires develop fast. The area coverage before the fire is threatening neighbor structures, i.e., theoretically dependent on the travel time squared, is further reduced. In combination with weather forecasts, especially wind speed and wind direction, the results of the present study may be used for better understanding potential conflagration risk. Being able to plan staffing and locations of fire trucks a few days in advance of a particularly challenging combination of dry indoor wood and high wind strength could turn out to be highly beneficial.

By studying the modeled results for the winter 2015/2016 in Haugesund, it may be seen that especially 8 to 22 January represented a period of high dry indoor climate wooden home fire risk. In this period, 9, 14 and 22 January were days with wind speeds of up to 15 m/s. A few days ahead of these dates, based on the weather forecast, the presented methodology could have been used to predict significantly higher conflagration risk these days compared to the year average. If ignited, wooden buildings would have produced excessive flames and firebrands, which could have been transported long distances involving new homes in the fire [21,22]. As is the case in many different cities, the fire station in Haugesund used to be in the city center, in close proximity to the wooden home area. It was, however, relocated in the 1970s to modern and more peripheral facilities. One may question whether the fire brigades could have arrived sufficiently early to prevent the loss of a number of wooden homes if a fire broke out in the densely built city center of Haugesund on 9, 14 or 22 January 2016. To evaluate this further would require modeling of the influence of wind speed and ambient relative humidity as well as the separation between the adjacent buildings.

Given that the indoor relative humidity can be recorded at a good accuracy, it is in principle always better to record it than to model it. There is an impressive work going on regarding development of new methods for recording the relative humidity, for example, see [54,55,56,57,58]. It is, therefore, quite likely that in the near future there will be very good RH sensors available at a reasonably low cost and without the need for time-consuming calibration. Then, modeling of the wood moisture content can be done quite precisely in the individual homes based on recorded indoor air RH. Based on current wood moisture content, the weather forecasts may then allow for a good fire risk prediction at least for a few days into the future.

The author hopes that the present work may be used in the future for cold climate fire risk assessments and, in combination with the wind conditions and the ambient relative humidity, provide information on conflagration risk. Being better prepared may help prevent losses as experienced in the Lærdalsøyri conflagration in 18–19 January 2014, and the WUI fire in Flatanger 10 days later [6,9,10,11]. Permanent manning of this particular fire station on high risk days, as experienced in January 2014, would have allowed for a significantly faster response in the case of a fire, and could have made it possible for the firefighters to contain the fire within the first building, thereby preventing the conflagration. This is in line with the fleet allocations, as suggested by Pérez et al. [24]; according to the seasons, although for shorter periods, or even single days, when the fire risk is especially high as assessed in the present study. Knowing the risk of fast fire development, accompanied by systems guiding the evacuation, as presented by Zhang et al. [59], may significantly contribute to lower consequences associated with fires in wooden buildings and densely built wooden town areas.

## 5. Conclusions

In order to assess the risk of fast fire development in wooden homes in cold climates, a methodology for modeling moisture content of indoor wooden home wall panels is outlined. The developed model was tested for four selected homes in Haugesund, Karmøy and Lærdal, Norway, during the winter of 2015/2016. By introducing a Bernoulli based air change rate, the model predicted the indoor relative humidity levels reasonably well and thereby provided information about expected indoor wooden wall panel moisture content. This information was used to assess single home fire risk, represented by an estimated time to flashover. In retrospect, it was found that three days of January 2016 was associated with very high conflagration risk in Haugesund due to a combination of dry indoor conditions and strong wind. This approach is novel and, based on weather forecasts, the concept may be further developed to provide information about single object fire risk and conflagration risk a few days into the future. This could enable risk based proactive emergency management increasing the likelihood of preventing wooden home fires to develop into conflagrations, i.e., town fires.

## References

[1] Park Y., Ryu J., Ryou H.S. (2019). Experimental Study on the Fire-Spreading Characteristics and Heat Release Rates of Burning Vehicles Using a Large-Scale Calorimeter. Energies.

[2] Novozhilov V. (2017). Fire Hazards of Some Modern Solid Fuels. Energies.

[3] Log T., Pedersen W.B., Moumets H. (2019). Optical Gas Imaging (OGI) as a Moderator for Interdisciplinary Cooperation, Reduced Emissions and Increased Safety. Energies.

[4] Huang H.S., Su C.H., Li C.B., Lin C.Y., Lin C.C. (2016). Enhancement of Fire Safety of an Existing Green Building due to Natural Ventilation. Energies.

[5] Losnegård G. (2013). Norske Ulykker og Katastrofar/Norwegian Accidents and Catastrophes.

[6] Log T. (2017). Indoor relative humidity as a fire risk indicator. Build. Environ..

[7] Pirsko A.R., Fons W.L. Frequency of Urban Building Fires as Related to Daily Weather Conditions. https://www.fs.fed.us/psw/publications/documents/cfres_series/cfres_itr_afswp866.pdf.

[8] Babrauskas V. (2003). Ignition Handbook.

[9] Log T. (2016). Cold Climate Fire Risk; A Case Study of the Lærdalsøyri Fire, January 2014. Fire Technol..

[10] (2014). DSB Report (2014) “Brannene I Lærdal, Flatanger op på Frøya vinteren 2014, Lærepunkter og anbefalinger/The Fires in Lærdal, Flatanger and Frøya the Winter 2014, Learning Points and Recommendations”.

[11] Steen-Hansen A., Bøe G.A., Hox K., Mikalsen R.F., Stensaas J.P., Storesund K. Evaluation of Fire Spread in the Large Lærdal Fire, January 2014. Proceedings of the 14th International Fire and Materials Conference and Exhibition.

[12] Delâge C. Rapport du Commissaire Aux Incendies du Québec. https://www.coroner.gouv.qc.ca/fileadmin/Coroners/Rapport_d_enquete_-_L_Isle-Verte.pdf.

[13] Metallinou M.M., Log T. (2017). Health Impacts of Climate Change-Induced Subzero Temperature Fires. Int. J. Environ. Res. Public Health.

[14] Manzello S.L., Blanchi R., Gollner M.J., Gorham D., McAllister S., Pastor E., Planas E., Reszka P., Suzuki S. (2018). Summary of workshop large outdoor fires and the built environment. Fire Saf. J..

[15] Manzello S.L., McAllister S., Suzuki S. (2018). Large outdoor fires and the built environment: Objectives and goals of permanent IAFSS working group. Fire Saf. J..

[16] Challands N. (2010). The Relationships Between Fire Service Response Time and Fire Outcomes. Fire Technol..

[17] Claridge E., Spearpoint M. (2013). New Zealand fire service response times to structure fires. Procedia Eng..

[18] Kraaijeveld A., Log T. Vertical Flame Spread in Wooden Corners as a Function of Fuel Moisture Content. Proceedings of the 15th International Conference Fire and Materials 2017.

[19] Kraaijeveld A., Gunnarshaug A., Schei B., Log T. Burning Rate and Time to Flashover in Wooden ¼ scale Compartments as a Function of Fuel Moisture Content. Proceedings of the 10th International Fire Science & Engineering Conference.

[20] Simpson W.T. (1998). Equilibrium Moisture Content of Wood in Outdoor Locations in the United States and Worldwide.

[21] Koo E., Pagni P.J., Weise D.R., Woycheese J.P. (2010). Firebrands and spotting ignition in large-scale fires. Int. J. Wildland Fire.

[22] Suzuki S., Manzello S.L. (2018). Characteristics of Firebrands Collected from Actual Urban Fires. Fire Technol..

[23] Metallinou M.M., Log T. (2018). Cold Climate Structural Fire Danger Rating System?. Challenges.

[24] Pérez J., Maldonado S., López-Ospina H. (2016). A fleet management model for the Santiago Fire Department. Fire Saf. J..

[25] Pietrzyk K. (2015). A systemic approach to moisture problems in buildings for mould safety modelling. Build. Environ..

[26] Kalamees T., Vali A., Kurik L., Napp M., Arumagi E., Kallavus U. (2016). The Influence of Indoor Climate Control on Risk for Damages in Naturally Ventilated Historic Churches in Cold Climate. Int. J. Archit. Herit..

[27] Pisello A.L., Cotana F., Nicolini A., Buratti C. (2014). Effect of dynamic characteristics of building envelope onthermal-energy performance in winter conditions: In field experiment. Energy Build..

[28] Vinha J., Korpi M., Salminen K., Kurnitski J., Kiviste M., Laukkarinen A. (2015). Airtightness of residential buildings in Finland. Build. Environ..

[29] Kotol M., Rode C., Clausen G., Nilsen T.R. (2014). Indoor environment in bedrooms in 79 Greenlandic households. Build. Environ..

[30] Vardoulakis S., Dimitroulopoulou C., Thornes J., Lai K., Taylor J., Myers I., Heaviside C., Mavrogianni A., Shrubsole C., Chalabi Z. (2015). Impact of climate change on the domestic indoor environment and associated health risks in the UK. Environ. Int..

[31] Kalamees T., Korpi M., Vinha J., Kurnitski J. (2009). The effects of ventilation systems and building fabric on the stability of indoor temperature and humidity in Finnish detached houses. Build. Environ..

[32] Tetens O. (1930). Uber einige meteorologische Begriffe. Z. Geophys..

[33] Jenssen J.A., Geving S., Johnsen R. Assessments on Indoor air Humidity in Four Different Types of Dwelling Randomly Selected in Trondheim, Norway. Proceedings of the 6th Symposium on Building Physics in the Nordic Countries.

[34] Kalamees T., Vinha J., Kurnitski J. (2006). Indoor Humidity Loads and Moisture Production in Lightweight Timber-frame Detached Houses. J. Build. Phys..

[35] Salin J.G. (2011). Inclusion of the Sorption Hysteresis Phenomenon in Future Drying Models. Some Basic Considerations. Maderas. Cienc. Tecnol..

[36] Funk M. (2014). Hysteretic moisture properties of porous materials: Part I: Thermodynamics. J. Build. Phys..

[37] Drysdale D. (2011). An Introduction to Fire Dynamics.

[38] (2016). ISO Reaction to Fire Tests—Room Corner Test for Wall and Ceiling Lining Products—Part 1: Test Method for a Small Room Configuration.

[39] Mortensen L.H. (2007). Hygrothermal Microclimate on Interior Surfaces of the Building Envelope. Ph.D. Thesis.

[40] Ramstad T.Ø., Hallquist Å. Scandinavian timber frame house construction technical design and future trends. Proceedings of the CIB World Building Congress.

[41] Baronas R., Ivanauskasa F., Juodeikienėc I., Kajalavičiusc A. (2001). Modelling of Moisture Movement in Wood during Outdoor Storage. Nonlinear Anal. Model. Control.

[42] Log T. (2018). Consumer Grade Weather Stations for Wooden Structure Fire Risk Assessment. Sensors.

[43] Rong L., Liu D.Z., Pedersen E.F., Zhang G.Q. (2014). Effect of climate parameters on air exchange rate and ammonia and methane emissions from a hybrid ventilated dairy cow building. Energy Build..

[44] Mortensen L.H., Rode C., Peuhkuri R. Full scale tests of moisture buffer capacity of wall materials. Proceedings of the 7th Symposium on Building Physics in the Nordic Countries.

[45] Svennberg K., Harderup L.E. Time-dependent moisture properties for plasterboard with surface coating. Proceedings of the 9th International Conference on Indoor Air Quality and Climate–Indoor Airil 2002.

[46] Rode C., Peuhkuri R., Mortensen L.H., Hansen K.K., Time B., Gustavsen A., Ojanen T., Ahonen J., Svennberg K., Harderup L.E. (2005). Moisture Buffering of Building Materials.

[47] Popper P., Niemz P., Croptier S. (2009). Adsorption and desorption measurements on selected exotic wood species. Analysis with the Hailwood-Horrobin model to describe the sorption hysteresis. Wood Res..

[48] Xie Y.J., Hill C.A.S., Jalaludin Z., Curling S.F., Anandjiwala R.D., Norton A.J., Newman G. (2011). The dynamic water vapour sorption behaviour of natural fibres and kinetic analysis using the parallel exponential kinetics model. J. Mater. Sci..

[49] Hill C.A.S., Keating B.A., Jalaludin Z., Mahrdt E. (2012). A rheological description of the water vapour sorption kinetics behaviour of wood invoking a model using a canonical assembly of Kelvin-Voigt elements and a possible link with sorption hysteresis. Holzforschung.

[50] Babrauskas V., Peacock R. (1992). Heat Release Rate: The Single Most Important Variable in Fire Hazard. Fire Saf. J..

[51] Averill J.D., Moore-Merrell L., Barowy A., Santos R., Peacock R., Notarianni K.A., Wissoker D., Robinson B. (2010). Report on Residential Fireground Field Experiments.

[52] Reglen D., Scheller D.S. (2016). Fire Department Turnout Times: A Contextual Analysis. J. Homel. Secur. Emerg. Manag..

[53] Upson R., Notarianni K. (2010). Quantitative Evaluation of Fire and EMS Mobilization Times.

[54] Boudaden J., Steinmaßl M., Endres H.E., Drost A., Eisele I., Kutter C., Müller-Buschbaum P. (2018). Polyimide-Based Capacitive Humidity Sensor. Sensors.

[55] Sun C., Shi Q., Yazici M.S., Lee C., Liu Y. (2018). Development of a Highly Sensitive Humidity Sensor Based on a Piezoelectric Micromachined Ultrasonic Transducer Array Functionalized with Graphene Oxide Thin Film. Sensors.

[56] Leal-Junior A., Frizera-Neto A., Marques C., Pontes M.J. (2018). Measurement of Temperature and Relative Humidity with Polymer Optical Fiber Sensors Based on the Induced Stress-Optic Effect. Sensors.

[57] Xu W., Shi J., Yang X., Xu D., Rong F., Zhao J., Yao J. (2017). Relative Humidity Sensor Based on No-Core Fiber Coated by Agarose-Gel Film. Sensors.

[58] Gaspar C., Olkkonen O., Passoja S., Smolander M. (2017). Paper as Active Layer in Inkjet-Printed Capacitive Humidity Sensors. Sensors.

[59] Zhang J., Guo J., Xiong H., Liu X., Zhang D. (2019). A Framework for an Intelligent and Personalized Fire Evacuation Management System. Sensors.

